# Response of circulating fatty acid binding protein 4 concentration to low-intensity acute aerobic exercise is amplified in an exercise duration-dependent manner in healthy men

**DOI:** 10.1186/s40101-024-00379-y

**Published:** 2024-12-20

**Authors:** Shigeharu Numao, Ryota Uchida, Masaki Nakagaichi

**Affiliations:** 1 Department of Sports and Life Sciences, National Institute of Fitness and Sports in Kanoya, 1 Shiromizu, Kanoya, Kagoshima, 891-2393 Japan; 2 Health and Sports Promotion Division, Sports Innovation Organization, National Institute of Fitness and Sports in Kanoya, 1 Shiromizu, Kanoya, Kagoshima, 891-2393 Japan

**Keywords:** Fatty acid-binding protein, Prolonged exercise, Sympathetic activity, Body fat

## Abstract

**Background:**

Circulating fatty acid-binding protein 4 (FABP4) influences cardiovascular disease and glucose metabolism. Acute aerobic exercise increases circulating FABP4 concentrations, but the factors underlying this effect in humans are unclear. We investigated the effect of exercise duration on circulating FABP4 concentrations in healthy men.

**Methods:**

This randomized crossover study enrolled healthy young men randomly assigned to two trials, short-duration (SE) and long-duration (LE) aerobic exercises trials. Both involved acute aerobic exercise followed by 60 min of bed rest. The exercise intensity was the same (40% peak oxygen uptake); however, the duration was 40 and 70 min for the SE and LE trials, respectively. Venous blood samples were collected to measure hormones, metabolites, and FABP4 concentrations.

**Results:**

Twelve healthy young men completed both trials. Changes in hormone levels did not differ significantly between the SE and LE trials (*p* > 0.05). However, the circulating FABP4 concentration increased significantly only in the LE trial immediately after exercise (*p* = 0.018). It increased significantly 30–60 min post-exercise in both the SE and LE trials (*p* < 0.018), with the extent of the increase being significantly higher in the LE trial than in the SE trial (*p* < 0.001). In each trial, the total incremental area under the curve of circulating FABP4 concentration was significantly positively correlated with body fat percentage (SE trial: *r*_s_ = 0.699, *p* = 0.019; LE trial: *r*_s_ = 0.643, *p* = 0.024).

**Conclusion:**

Our findings suggest that exercise duration is associated with the magnitude of increased FABP4 secretion into the blood circulation. Body fat accumulation may also be involved in the magnitude of FABP4 secretion induced by acute aerobic exercise.

**Trial registration:**

The study was pre-registered with the University Hospital Medical Information Network Center (UMIN), a clinical trial registration system (ID: UMIN000051068).

**Supplementary Information:**

The online version contains supplementary material available at 10.1186/s40101-024-00379-y.

## Introduction

Fatty acid-binding protein 4 (FABP4), also known as adipocyte FABP or adipose protein 2, is crucial in regulating lipid traffic and cellular responses [1]. FABP4 is secreted from adipocytes [2, 3] and endothelial cells [4] into the blood circulation and excreted via the kidneys [5, 6]. An increase in circulating FABP4 levels is generally considered to have adverse health effects. FABP4 is recognized as a pro-inflammatory adipokine [7]. Additionally, circulating FABP4 concentrations are associated with various disorders, such as atherosclerotic lesions, insulin resistance, diabetes, dyslipidemia, and cancer [1, 8, 9]. Several studies also have shown that circulating FABP4 increases hepatic glucose production, attenuates glucose disposal [2, 10], elevates insulin secretion [11], and decreases contraction amplitude in isolated cardiomyocytes [12]. Therefore, it is essential to understand the factors influencing circulating FABP4 levels to prevent various diseases and disorders.

The mechanisms underlying FABP4 secretion by adipocytes have been investigated. FABP4 secretion from adipocytes is enhanced by β-adrenergic stimulation [2, 3, 13–15]. In particular, the activity of adipocyte triglyceride lipase (ATGL) and hormone-sensitive lipase (HSL), enzymes involved in lipolysis, was found to play an important role in FABP4 secretion [15]. However, recent studies suggest that sympathetic signaling has a stronger influence on FABP4 secretion than the activity of these enzymes in vivo [13].

Acute aerobic exercise appears to alter FABP4 secretion [16–18]. Since β-adrenergic stimulation is enhanced during acute aerobic exercise [19], circulating FABP4 concentration appears to be increased by acute aerobic exercise. Acute short-duration exercise at high and maximal intensity increases circulating FABP4 concentrations immediately after exercise [16, 18]. The increase in circulating FABP4 concentrations positively correlates with increased adrenaline and noradrenaline concentrations [16]. However, acute aerobic exercises at low and moderate intensity do not alter circulating FABP4 concentrations immediately after exercise [17]. Instead, circulating FABP4 concentrations increase 30–60 min post-exercise of acute low- and moderate-intensity aerobic exercises [17, 20].

Given that the circulating FABP4 concentration remains unchanged at rest [17, 21], the observed increase in FABP4 concentrations after 30 min post-exercise following low- and moderate-intensity aerobic exercise [17, 20] is assumed to be a response induced by aerobic exercises. This may be caused by the delayed acceleration of FABP4 secretion in response to physiological stimuli triggered by low- and moderate-intensity aerobic exercises.

If FABP4 secretion from adipocytes is delayed in response to low- and moderate-intensity acute aerobic exercise, FABP4 concentrations are likely to increase immediately after exercise as the exercise duration extends. Furthermore, FABP4 concentrations may cumulatively rise following long-duration acute aerobic exercise. Thus, exercise duration may significantly influence FABP4 concentration responses induced by low- and moderate-intensity acute aerobic exercise. In our previous study, we examined the effects of acute aerobic exercise at low (40% peak oxygen uptake [VO_2_peak]) and moderate (60%VO_2_peak) intensities on circulating FABP4 concentrations [17]. Energy expenditure was matched between exercises to isolate the effects of exercise intensity on circulating FABP4 concentrations. Consequently, although exercise durations differed (40 min for low-intensity and 25 min for moderate-intensity acute aerobic exercise), changes in circulating FABP4 concentrations did not differ between the two intensity levels. However, these findings do not necessarily clarify the independent effect of exercise duration on circulating FABP4 concentrations, as the influence of exercise intensity cannot be entirely excluded. Therefore, it is essential to investigate the response of circulating FABP4 concentrations during exercises of identical intensity but different durations.

To identify the factors associated with increased circulating FABP4 concentration immediately after aerobic exercise and post-exercise, we investigated the effects of exercise duration on circulating FABP4 concentrations immediately after acute aerobic exercise and post-exercise at low intensity in healthy men. We hypothesized that the circulating FABP4 concentration increases immediately after acute aerobic exercise at a longer duration and low intensity, and it is higher after acute aerobic exercise at a longer duration and low intensity than at a short duration and low intensity.

## Methods

### Ethics approval

The study adhered to the principles outlined in the Declaration of Helsinki and was approved by the Ethics Committee of the National Institute of Fitness and Sports in Kanoya (approval number: 22–1-72). The study was preregistered with the University Hospital Medical Information Network Center (UMIN), a clinical trial registration system (ID: UMIN000051068).

### Study design and participants

This was a randomized crossover trial. The enrollment period was between April 2023 and May 2023. The experiments were conducted between June 2023 and August 2023, including preliminary testing and two main trials. After a detailed explanation of the purpose, design, protocol, and potential risks of the study, each of the 12 healthy young men provided written informed consent. The exclusion criteria were as follows: (1) women (biological sex), (2) age < 18 or > 40 years, (2) regular exercise habits, (3) taking medications known to affect lipid and carbohydrate metabolism, and (4) current smoking. The flowchart of participants is shown in Figure S1.

### Preliminary testing

Height was measured using a stadiometer with a 0.1-cm accuracy. Weight, fat mass, fat-free mass, and skeletal muscle mass were measured with a 0.1-kg precision using a dual-frequency body composition monitor (Inbody770, InBody Japan Inc., Tokyo, Japan). Body mass index was calculated as weight in kilograms divided by the square of height in meters. Resting blood pressure was measured using an automatic sphygmomanometer (HEM-1040, Omron Corp., Kyoto, Japan) after participants rested for 15 min. Moderate- and vigorous-intensity physical activity levels were assessed using the Global Physical Activity Questionnaire [22]. Aerobic capacity represented by peak oxygen uptake (VO_2_peak) was determined using an incremental exercise protocol in which, after a brief warm-up at 30 W on a cycle ergometer (Aerobike 75XLIII, Konami Sports Life, Kanagawa, Japan), power was increased by 15 W every 1 min. During the test, ventilation and gas exchange were measured using indirect calorimetry (K5, COSMED, Rome, Italy). The criteria for reaching VO_2_peak have been described in detail previously [23]. The highest VO_2_ value achieved over 30 s was considered the VO_2_peak.

### Study procedure

**Fig. 1:**
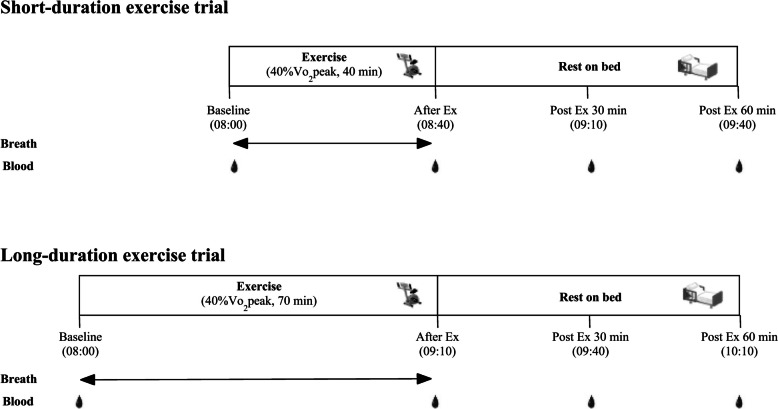
Experimental protocol

The study comprised two experimental trials with a minimum interval of 1 week between trials to mitigate any potential carry-over effects. Participants were randomly assigned to one of the two trials in a counterbalanced manner. Randomization was performed using a computer software formula (Microsoft Excel, Microsoft, Tokyo, Japan). The principal investigator enrolled the participants and assigned allocation sequences. The protocol is illustrated in Fig. 1. The two trials were as follows: (1) short-duration aerobic exercise + rest (SE) and (2) long-duration aerobic exercise + rest (LE). The participants arrived at our laboratory at 7:40 AM after fasting overnight for at least 12 h and drinking only water. Upon arrival, each participant rested supine for 15 min, after which a fasting venous blood sample (baseline) was collected. Blood pressure was measured using an automatic sphygmomanometer (HEM-1040, Omron Corp., Kyoto, Japan), and body composition was assessed using a body composition analyzer (Inbody770, InBody Japan Inc., Tokyo, Japan).

The participants then completed a cycle exercise for 40 and 70 min in SE and LE trials, respectively. Both trials were conducted in a fasted state, with participants abstaining from breakfast. The exercise intensity was set to a workload corresponding to 40% of VO_2_peak. The exercise duration and intensity were determined based on our previous studies [17, 20]. Especially, the exercise duration for LE was extended by 30 min (40 min + 30 min = 70 min), because FABP4 increases after at least 30 min post-exercise following 40 min aerobic exercise [17, 20]. After both exercise sessions, participants rested supine on a bed for 60 min. The arrival time, blood sampling, and start of exercise were synchronized for each participant between the two trials to exclude possible effects of circadian rhythm. During both trials, participants were allowed to drink mineral water as needed.

Indirect calorimetry (K5, COSMED, Rome, Italy) was used to measure ventilation and gas exchange during aerobic exercise at 9–10, 19–20, 29–30, 39–40 min for SE trial, and 9–10, 19–20, 29–30, 39–40, 49–50, 59–60, 69–70 min for LE trial. Heart rate (HR) was monitored continuously during the trials using a wrist device (Polar A370, Polar Japan, Tokyo, Japan). Venous blood samples were collected from each participant immediately after exercise and at 30- and 60-min intervals postexercise. To ensure consistency, the participants received identical meals the day before each trial and were instructed to refrain from strenuous exercise and physical activity 24 h before each trial. The participants verbally confirmed compliance with the conditions before each trial.

### Blood sampling and analysis

Blood samples were collected in the following tube types: (1) 6-mL tubes containing thrombin, (2) 7-mL tubes containing sodium ethylenediaminetetraacetic acid (EDTA), (3) 2-mL tubes containing potassium EDTA, and (4) 2-mL tubes containing sodium fluoride, heparin sodium, and sodium EDTA. The 6-ml tubes were centrifuged at 3000 × *g* for 10 min at room temperature 30 min after collection. The 7-mL tubes were centrifuged at 3000 × *g* for 10 min at 4 °C immediately after collection. Serum and plasma samples were transferred to plastic tubes and immediately stored at − 80 °C until further analysis. The blood in the 2-mL tubes was used to measure hemoglobin, hematocrit, and glucose levels.

At baseline, blood samples were used to measure the following parameters: plasma adrenaline and noradrenaline, glucose concentrations, serum insulin, cortisol, creatinine (Cre), free fatty acid (FFA), glycerol, FABP4 concentrations, blood total cholesterol (TC), high-density lipoprotein cholesterol (HDLC), triglyceride (TG) concentrations, and hemoglobinA1c (HbA1c). Subsequent blood samples, excluding baseline samples, were used to determine plasma adrenaline and noradrenaline levels as well as serum insulin, cortisol, Cre, glucose, FFA, glycerol, and FABP4 concentrations.

Plasma adrenaline and noradrenaline concentrations were determined using high-performance liquid chromatography (Tosoh Corporation, Tokyo, Japan). Plasma glucose concentration was analyzed using an enzymatic method (Hitachi Chemical Diagnostics Systems Corporation, Tokyo, Japan). Serum insulin level was measured using a chemiluminescent immunoassay (Abbott Japan, Tokyo, Japan). Serum cortisol concentration was analyzed using an electrochemiluminescent immunoassay (Roche Diagnostics, Tokyo, Japan). Serum Cre concentration was determined using the endogenous creatinine elimination reaction method (Kanto Chemical Corporation, Tokyo, Japan). Blood TC, HDLC, TG, and HbA1c levels were measured using an automatic analyzer (Cobas b 101 plus, Roche Diagnostics, Tokyo, Japan). Serum FFA concentration was measured using an enzymatic method (FUJIFILM Wako Pure Chemical Corp., Osaka, Japan). Serum glycerol concentration was analyzed using a coupled enzymatic reaction (Cayman Chemical, MI, USA). Serum FABP4 concentration was measured using an enzyme-linked immunosorbent assay kit (R&D Systems Inc., MN, USA). To exclude inter-assay variability, samples from each participant were analyzed in the same run. The intra-assay coefficient of variation of the analysis for FFA, glycerol, and FABP4 concentrations was < 5.0%.

The estimated glomerular filtration rate (eGFR) was calculated using the equation for adult Japanese men (194 × creatinine concentration (mg/dL)^−1.094^ × age (years)^−0.287^). Serum glucose concentration was measured using an enzymatic method (Hitachi Chemical Diagnostics Systems Corporation, Tokyo, Japan). Low-density lipoprotein cholesterol concentration was estimated using the Friedewald equation [24]. Hemoglobin and hematocrit levels were measured using an automatic analyzer (MEK-1301, Nihon Kohden Corp., Tokyo, Japan).

### Substrate oxidation

Energy expenditure (EE) and carbohydrate and fat oxidation rates were calculated from VO_2_, carbon dioxide output_,_ and the respiratory exchange ratio (RER) [25].

### Statistical analysis

The primary outcome was the change in the FABP4 concentration in the SE and LE trials. The Kolmogorov–Smirnov and Levene’s tests were used to confirm normality and homoscedasticity, respectively. Variables that were not normally distributed were log-transformed before statistical analysis. A paired t-test was used to determine the differences in physical characteristics, exercise intensity (HR, VO_2_, and %VO_2_), EE, RER, and substrate oxidation during exercise between the SE and LE trials. A two-way repeated-measures analysis of variance (trial × time) was performed to examine the changes in blood parameters between the two trials. When a significant interaction was observed, a Bonferroni post hoc analysis was conducted to determine the differences between trials at a specific time and between time points in each trial. The effect size (ES) was calculated as Cohen’s *d* (small ≥ 0.20, medium ≥ 0.50, or large ≥ 0.80) for the post hoc test. To assess the changes in the concentrations of hormones and metabolites (adrenaline, noradrenaline, cortisol, insulin, glucose, FFA, glycerol, and FABP4) during the SE and LE trials, the incremental area under the curve (iAUC) was calculated using the trapezoidal rule. Since the exercise duration differed between the SE and LE trials, the iAUC was adjusted for time (min). Spearman’s rank correlation coefficients were calculated to estimate (1) the relationship between the total iAUC of FABP4 concentration, the iAUC from baseline to immediately after exercise for hormones and metabolites, and body composition in each trial, (2) the relationship between the changes in FABP4 concentration, and the changes in hormones and metabolites from baseline to immediately after exercise in each trial and (3) the relationship between the changes in FABP4 concentration, and the fat oxidation and percentage of fat oxidation to energy expenditure during aerobic exercise in each trial.

All data are expressed as mean ± standard deviation (SD) or median. Blood parameters were adjusted according to changes in plasma volume [26]. The sample size was determined using G*Power version 3.1.3 [27], based on the ES (*f* = 0.25) of the change in FABP4 concentration during exercise [17]. The calculation indicated that a minimum sample size of nine was required to achieve a statistical power of approximately 80% with a significance level of 0.05. Statistical analyses were performed using SPSS version 28 software (IBM Corporation, Armonk, NY, USA). Statistical significance was set at *p* < 0.05.

## Results

### Characteristics of the participants

Twelve healthy young men completed SE and LE trials. Physical characteristics did not significantly differ between the SE and LE trials (Table 1). No adverse events were observed. The body weight of the participants remained stable over the previous 6 months. None of the participants had orthopedic conditions or a history of metabolic, cardiovascular, or gastrointestinal disease. All participants were non-smokers and had not taken any medications or supplements known to affect lipid or carbohydrate metabolism.

**Table 1:** Physical characteristics of participants

	SE trial	LE trial
Age (years)	22.1 ± 1.6 (19–24)	
Height (cm)	174.8 ± 5.8 (162.3–181.2)	
Weight (kg)	68.7 ± 5.2 (60.5–77.1)	68.6 ± 4.8 (60.9–77.2)
BMI (kg/m^2^)	22.5 ± 1.0 (20.6–24.0)	22.4 ± 1.0 (20.1–24.1)
*Body composition*		
%Fat (%)	14.9 ± 3.6 (10.3–21.1)	14.7 ± 3.8 (9.8–21.2)
Fat mass (kg)	10.2 ± 2.5 (6.9–13.7)	10.1 ± 2.7 (6.4–14.3)
Skeletal muscle mass (kg)	55.3 ± 4.8 (48.4–63.6)	55.3 ± 4.6 (48.8–62.9)
*Blood pressure*		
Systolic blood pressure (mmHg)	107.1 ± 8.7 (91–117)	107.0 ± 9.3 (92–122)
Diastolic blood pressure (mmHg)	63.9 ± 7.2 (49–74)	64.0 ± 9.5 (52–73)
*Health-related blood parameters*		
TC (mg/dL)	160.0 ± 26.5 (123–212)	156.9 ± 22.8 (122–191)
TG (mg/dL)	76.3 ± 16.8 (50–102)	77.4 ± 14.4 (54–93)
HDLC (mg/dL)	54.8 ± 11.6 (36–77)	54.2 ± 10.0 (38–76)
LDLC (mg/dL)	89.6 ± 21.1 (52–121)	87.3 ± 16.0 (55–120)
Hemoglobin A1_C_ (%)	5.2 ± 0.2 (4.8–5.5)	5.2 ± 0.2 (4.7–5.7)
Creatinine (mg/dL)	0.89 ± 0.11 (0.72–1.09)	0.92 ± 0.14 (0.63–1.18)
eGFR (mL/min/1.73 m^2^)	92.1 ± 14.0 (72.7–116.0)	89.9 ± 16.8 (66.7–134.2)
*Aerobic capacity*		
Maximal HR (beat/min)	188.8 ± 6.8 (178–201)	
Maximal load (watts)	235.0 ± 24.2 (195–270)	
VO_2_peak (ml/kg/min)	44.7 ± 5.5 (35.7–54.8)	
VO_2_peak (ml/min)	3097.2 ± 370 (2661–3720)	
*Physical activity*		
MPA (min/week)	90 (0–600)	
VPA (min/week)	90 (0–180)	

Values are presented as mean ± SD (range) or median (range)

*SE* short-duration exercise, *LE* long-duration exercise, *BMI* body mass index, *%fat* percentage of fat, *TC* total cholesterol, *TG* triglycerides, *HDLC* high-density lipoprotein cholesterol, *LDLC* low-density lipoprotein cholesterol, *eGFR* estimated glomerular filtration rate, *HR* heart rate, *VO*_*2*_*peak* peak oxygen uptake, *MPA* moderate-intensity physical activity, *VPA* vigorous-intensity physical activity

### Exercise intensity and substrate oxidation

Exercise intensity and substrate oxidation parameters in the SE and LE trials are shown in Table 2. The mean HR, VO_2_, and %VO_2_ during exercise did not differ significantly between the SE and LE trials (all *p* > 0.05). The mean RER during exercise was lower in the LE trial than in the SE trial (*p* = 0.052, ES = 0.63), indicating a significantly higher percentage of fat oxidation in the LE trial than in the SE trial (*p* = 0.040, ES = 0.67).

**Table 2:** Heart rate, workload, absolute and relative oxygen consumption, respiratory exchange ratio, and substrate oxidation during aerobic exercise in the SE, and LE trials

	SE	LE	*p* value	ES
HR (beat/min)	108 ± 9	112 ± 9	0.134	0.43
Workload (watts)	64.0 ± 12.7			
VO_2_ (mL/min)	1208 ± 177	1206 ± 150	0.987	0.01
%VO_2_ (mL/min)	39.1 ± 5.0	39.0 ± 3.5	0.961	0.01
RER	0.95 ± 0.08	0.90 ± 0.05	0.052	0.63
EE (kcal)	239.6 ± 9.9	412.7 ± 14.3	< 0.001	5.09
Carbohydrate oxidation (g)	45.5 ± 11.2	65.2 ± 18.9	0.007	0.96
Fat oxidation (g)	6.4 ± 4.9	16.9 ± 9.7	0.001	1.26
% Carbohydrate oxidation (%)	76.6 ± 5.1	64.0 ± 5.3	0.046	0.65
% Fat oxidation (%)	23.4 ± 5.1	36.3 ± 5.3	0.040	0.67

Values are presented as means ± SD

*HR* heart rate, *Vo*_*2*_ oxygen uptake, *RER* respiratory exchange ratio, *EE* energy expenditure, *SE* short-duration exercise trial, *LE* long-duration exercise trial, *ES* effect size

### Hormones

The hormone responses in the SE and LE trials are shown in Table 3. No significant trial × time interactions were observed for adrenaline and noradrenaline concentrations between the SE and LE trials (*p* = 0.075 and 0.124, respectively). Adrenaline and noradrenaline concentrations increased immediately after exercise (*p* < 0.001, ES = 1.71–2.46 and *p* = 0.001, ES = 2.20–2.26, respectively) and returned to baseline levels 30 and 60 min post-exercise in the SE and LE trials. There was no significant trial × time interaction in cortisol concentrations between the SE and LE trials (*p* = 0.971). Similarly, cortisol concentrations decreased significantly in the SE and LE trials (*p* < 0.02, ES = 1.40–2.54). No significant trial × time interaction was observed in insulin concentration between the SE and LE trials (*p* = 0.359); however, the main effects of trial and time were significant (trial: *p* = 0.018 and time: *p* < 0.001). Insulin concentrations decreased immediately after exercise in both trials (*p* = 0.009, ES = 1.12).

**Table 3:** The response of hormones to the SE and LE trials

	Baseline	Immediately after Ex	Post-Ex 30 min	Post-Ex 60 min	*p* value		
					Interaction	Trial	Time
Adrenaline (pg/ml)							
SE trial	23.3 ± 9.8	37.8 ± 15.3^b^	22.2 ± 9.5	19.6 ± 9.3	0.075	0.109	< 0.001
LE trial	25.8 ± 10.8	50.7 ± 17.9^b^	23.6 ± 8.3	21.9 ± 6.3			
Noradrenaline (pg/ml)							
SE trial	243.3 ± 85.0	357.4 ± 91.7^b^	166.8 ± 39.7	176.3 ± 59.1	0.124	0.352	< 0.001
LE trial	252.5 ± 79.8	408.3 ± 100.4^b^	186.9 ± 90.8	169.1 ± 65.0			
Cortisol (µg/dl)							
SE trial	14.2 ± 2.8	9.8 ± 2.0^b^	8.8 ± 2.3^b^	8.1 ± 2.7^b^	0.971	0.459	< 0.001
LE trial	13.7 ± 2.9	9.5 ± 3.3^b^	8.7 ± 3.5^b^	7.6 ± 2.7^b^			
Insulin (µU/ml)							
SE trial	5.8 ± 2.1^a^	3.7 ± 1.1^a, b^	4.1 ± 1.5^a^	3.8 ± 1.4	0.359	0.018	< 0.001
LE trial	4.6 ± 1.2	2.8 ± 1.0^b^	3.2 ± 1.2	3.3 ± 1.1			

Data are presented as mean ± SD. *SE* short-duration exercise, *LE* long-duration exercise, *Ex* exercise

^a^Significantly different between the trials at the same time point

^b^Significantly different from baseline in each trial

### Metabolites

The metabolite responses in the SE and LE trials are shown in Table 4. A significant trial × time interaction was observed in glycerol concentrations between the SE and LE trials (*p* = 0.015). Glycerol concentrations increased significantly immediately after exercise in the SE and LE trials (*p* < 0.001, ES = 1.20–1.55) and were significantly higher in the LE trial than in the SE trial (*p* = 0.022, ES = 0.76). Glycerol concentrations returned to baseline 30 and 60 min post-exercise in both trials. In contrast, no significant trial × time interaction was observed for FFA concentration between the SE and LE trials (*p* = 0.251). Still, the main effects of trial and time were significant (trial: *p* = 0.002, ES = 0.70, and time: *p* = 0.002, ES = 0.91). No significant trial × time interaction was observed for glucose concentration between the SE and LE trials (*p* = 0.121). Glucose concentration decreased significantly immediately after exercise (*p* < 0.001, ES = 1.91) and returned to baseline 30 and 60 min post-exercise in both trials. No significant trial × time interaction was observed for Cre concentration between the SE and LE trials (*p* = 0.534). In each trial, Cre concentrations were significantly higher post-exercise than immediately after exercise (*p* < 0.001, ES = 1.87–1.94), but those values were not significantly different from the baseline (*p* > 0.171). In each trial, the eGFR 30 and 60 min post-exercise was significantly lower than that at baseline and immediately after exercise (*p* < 0.001, ES = 0.74–2.02).

**Table 4:** The response of metabolites and renal functions to the SE and LE trials

	Baseline	Immediately after Ex	Post-Ex 30 min	Post-Ex 60 min	*p* value		
					Interaction	Trial	Time
Glycerol (mg/dl)							
SE trial	4.9 ± 1.4	6.5 ± 2.2^a,b^	4.4 ± 1.2^a^	4.5 ± 1.2	0.015	0.058	< 0.001
LE trial	5.5 ± 1.9	8.9 ± 2.6^b^	5.3 ± 1.3	4.5 ± 0.8			
FFA (mmol/ml)							
SE trial	0.40 ± 0.16	0.46 ± 0.20	0.50 ± 0.17	0.36 ± 0.18	0.251	0.002	0.002
LE trial	0.47 ± 0.20	0.65 ± 0.14	0.61 ± 0.17	0.56 ± 0.15			
Glucose (mg/dl)							
SE trial	86.9 ± 5.0	79.2 ± 3.5	89.1 ± 5.2	89.0 ± 5.3	0.121	0.340	< 0.001
LE trial	86.2 ± 5.9	76.5 ± 6.6	85.0 ± 8.7	88.0 ± 7.7			
Creatinine (mg/dl)							
SE trial	0.89 ± 0.11	0.87 ± 0.10	0.95 ± 0.11	0.95 ± 0.12	0.534	0.563	< 0.001
LE trial	0.92 ± 0.14	0.88 ± 0.10	0.93 ± 0.13	0.97 ± 0.12			
eGFR (ml/min/1.73 m^2^)							
SE trial	93.6 ± 13.7	95.2 ± 12.2	86.8 ± 12.1	87.2 ± 14.1	0.448	0.641	< 0.001
LE trial	91.1 ± 16.5	94.9 ± 12.3	88.5 ± 15.5	84.7 ± 12.9			

Data are presented as mean ± SD. *SE* short-duration exercise, *LE* long-duration exercise, *Ex* exercise, *FFA* free fatty acid, *eGFR* estimated globular filtration rate

^a^Significantly different between the trials at the same time point

^b^Significantly different from baseline in each trial

### FABP4 concentration

**Fig. 2:**
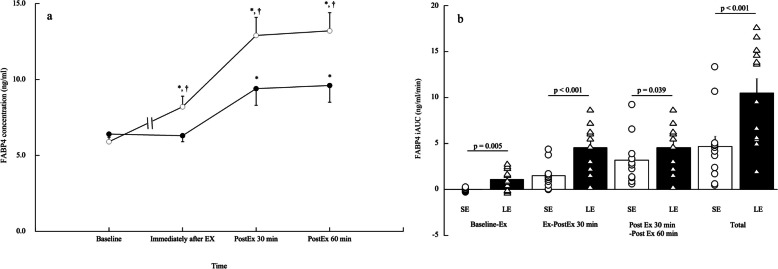
Response (**a**) and iAUC (**b**) of circulating FABP4 concentrations in the SE and LE trials. **a** Values are shown as mean ± standard error. Solid circles and open circles are presented as the SE and LE trials, respectively. * vs baseline, † vs SE trial at the same time point. **b** Values are shown as mean ± standard error. Open circle and open triangle plots are presented as individual values in the SE and LE trials, respectively

The responses and iAUCs of FABP4 concentrations are shown in Fig. 2. A significant trial × time interaction was observed for FABP4 concentrations between the SE and LE trials (*p* < 0.001; Fig. 2a). Specifically, FABP4 concentrations increased significantly immediately after exercise in the LE trial (*p* = 0.018, ES = 1.06) but remained unchanged immediately after exercise in the SE trial (*p* = 1.000). Furthermore, FABP4 concentrations significantly increased 30–60 min post-exercise in both trials (*p* < 0.018, ES = 1.28–1.37), but the magnitude of the increase in FABP4 concentrations was significantly higher in the LE trial than in the SE trial (*p* < 0.001, ES = 1.21–1.42). The iAUCs during and after exercise were significantly higher in the LE trial than in the SE trial (*p* ≤ 0.039, ES = 0.66–1.54; Fig. 2b). The total iAUC was also higher in the LE trial than in the SE trial (*p* < 0.001, ES = 1.63).

### The relationship between total iAUC of FABP4 concentration and iAUC from baseline to immediately after exercise for hormones, metabolites, and body composition

**Fig. 3:**
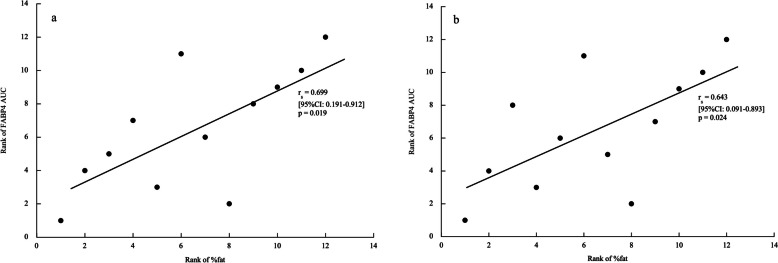
Bivariate relationships between the percentage of body fat and iAUC of FABP4 concentration in the SE (**a**) and LE (**b**) trials

The Spearman rank correlation coefficients between the total iAUC of FABP4 concentration and iAUC from baseline to immediately after exercise for hormones, metabolites, and body composition are presented in Table 5. In both trials, there were no significant correlations between the total iAUC of FABP4 concentration and iAUC from baseline to immediately after exercise for hormones or metabolites. In contrast, a significant positive correlation was observed between total iAUC of FABP4 concentration and fat percentage in both trials (SE trial: *r*_s_ = 0.699, *p* = 0.019; LE trial: *r*_s_ = 0.643, *p* = 0.024; Fig. 3a, b).

**Table 5:** The Spearman rank correlation coefficients between the total iAUC of FABP4 concentration, the iAUC from baseline to immediately after exercise for hormones and metabolites, and body composition

	Adrenaline^a^	Noradrenaline^a^	Cortisol^a^	Insulin^a^	Glucose^a^	FFA^a^	Glycerol^a^	BMI	FM	SMM
*SE trial*										
*r*_s_	0.049	0.231	− 0.042	0.091	− 0.259	0.098	0.210	0.406	0.622	− 0.517
*p*-value	0.880	0.471	0.897	0.779	0.417	0.762	0.513	0.191	0.031	0.085
*LE trial*										
*r*_s_	− 0.427	− 0.231	− 0.084	0.385	0.336	0.462	0.049	0.490	0.545	− 0.517
*p*-value	0.167	0.471	0.795	0.217	0.286	0.131	0.880	0.106	0.067	0.085

*FFA* free fatty acid, *BMI* body mass index, *FM* fat mass, *SMM* skeletal muscle mass, *SE* short-duration exercise, *LE* long-duration exercise

^a^Incremental area under the curve from baseline to immediately after exercise

### The relationship between the changes in FABP4 concentration and the changes in hormones, and metabolites from baseline to immediately after exercise

The Spearman rank correlation coefficients between the changes in FABP4 concentration and the changes in hormones and metabolites from baseline to immediately after exercise are presented in Table S1. In both trials, there were no significant correlations between the changes in FABP4 concentration and the changes in hormones and metabolites from baseline to immediately after exercise.

### The relationship between the changes in FABP4 concentration and fat oxidation and the percentage of fat oxidation to energy expenditure during aerobic exercise

The Spearman rank correlation coefficients between the changes in FABP4 concentration and fat oxidation and the percentage of fat oxidation to energy expenditure during aerobic exercise are presented in Table S2. In both trials, no significant correlations were observed between the changes in FABP4 concentration and fat oxidation and the percentage of fat oxidation to energy expenditure during aerobic exercise.

## Discussion

The primary findings of this study revealed that the increase in circulating FABP4 concentrations is amplified immediately after and post-exercise of low-intensity aerobic exercise, depending on the duration of exercise. These results support our hypotheses. Although no correlation was observed between the increase in circulating FABP4 concentrations and the changes in hormone and metabolite concentrations, the body fat percentage was associated with increased circulating FABP4 concentrations. These findings suggest that the FABP4 secretory response to exercise is time-consuming. Additionally, the accumulation of exercise duration and body fat is likely one of the potential factors influencing the magnitude of FABP4 secretion by acute aerobic exercise.

The increase in circulating FABP4 concentration immediately after acute exercise and post-exercise has been documented [16–18, 20], and our results are consistent with this phenomenon. Vigorous-to-maximal-intensity exercise increases circulating FABP4 concentrations immediately after exercise [16, 18]. In contrast, low- to moderate-intensity aerobic exercise does not increase circulating FABP4 concentrations immediately after exercise; instead, an increase is observed only from 10 min after the end of exercise [16, 17, 20]. Increased circulating FABP4 concentrations generally result from decreased FABP4 clearance from the circulation, increased FABP4 secretion into the circulation, or both. Circulating FABP4 is ultimately excreted via the kidneys [5, 6]. Therefore, it is likely that reduced excretion of FABP4 from the circulation is one of the factors increasing circulating FABP4 concentrations. However, despite similar Cre and eGFR responses immediately after exercise and post-exercise between the SE and LE trials, circulating FABP4 concentrations were higher in the LE trial than in the SE trial immediately after exercise and during the post-exercise period. This suggests that the difference in circulating FABP4 concentrations between the LE and SE trials cannot be explained by reduced clearance of FABP4 alone. It is, therefore, reasonable to assume that FABP4 secretion induced by aerobic exercise was greater in the LE trial than in the SE trial. In the LE trial, FABP4 secretion may have increased further during exercise and post-exercise, which could explain the observed differences in FABP4 concentrations between the LE and SE trials, not only immediately after exercise but also in the post-exercise.

Since increased circulating FABP4 concentrations due to lipolysis in vivo are derived from adipocytes [4], increased circulating FABP4 concentrations induced by acute exercise may be attributed to increased FABP4 secretion from adipocytes. In short, acute aerobic exercise likely triggers FABP4 secretion, particularly from adipocytes. Although the mechanisms behind increased FABP4 secretion from adipocytes by acute exercise are not yet fully understood, increased sympathetic activity or lipolysis itself during acute aerobic exercise may contribute to this secretion. Several studies have investigated the mechanisms underlying adipocyte FABP4 secretion in vivo, in vitro, and ex vivo [2–4, 14, 15]. In a previous ex vivo study, pharmacological inhibition and genetic deficiency of ATGL and HSL attenuated FABP4 secretion from adipocytes [15], suggesting that lipolysis is required for FABP4 secretion from adipocytes. Conversely, a recent in vivo study demonstrated that circulating corticosterone concentrations, which can enhance sympathetic activity [28, 29], are positively correlated with circulating FABP4 concentrations, and blocking or reducing sympathetic signaling decreases FABP4 secretion in adipocyte-specific AGTL-deficient mice [4], implying that sympathetic activity is essential for FABP4 secretion from adipocytes. Thus, the exact mechanisms of adipocyte FABP4 secretion remain unclear. In this study, adrenaline and noradrenaline concentrations, indicators of sympathetic activity, increased similarly in both the SE and LE trials, with no observed correlations between the increased circulating FABP4 concentration and increased adrenaline or noradrenaline levels. Glycerol concentration, a marker of lipolysis, increased during acute aerobic exercise in both the SE and LE trials, with a greater increase in the LE than in the SE trial. Thus, the contribution of lipolysis to FABP4 secretion following acute aerobic exercise may exceed that of sympathetic stimulation. However, a previous study indicated that inhibiting lipolysis by carbohydrate intake does not suppress the increase in circulating FABP4 levels induced by acute exercise [20]. Further studies using more direct markers, such as isotopic tracers, are needed to clarify the roles of sympathetic stimulation and lipolysis in exercise-induced FABP4 secretion.

Previous studies have reported increased circulating FABP4 concentrations after low- to moderate-intensity aerobic exercise [17], suggesting that increased FABP4 secretion induced by low-intensity aerobic exercise is delayed. In this study, circulating FABP4 concentrations increased 30- and 60-min post-exercise in the SE trial and increased immediately after exercise, 30- and 60-min post-exercise in the LE trial. Notably, the circulating FABP4 concentration immediately after exercise in the LE trial was comparable to that observed 30 min post-exercise in the SE trial. Based on the timing and magnitude of these increases in FABP4, we speculate that FABP4 secretion induced by low-intensity aerobic exercise is related to the time that elapsed after the onset of exercise. In the SE trial, FABP4 increased at 70 and 100 min from the start of exercise, while in the LE trial, the increase was observed at 70, 100, and 130 min. Thus, FABP4 appears to increase at consistent intervals times from the start of exercise.

Insulin suppresses FABP4 secretion in adipocytes [2, 3], in mice [10], and in humans [3, 10]. Therefore, the potential effect of insulin on FABP4 concentrations should be considered. In this study, insulin concentrations were significantly higher at baseline in the SE than in the LE trials. However, baseline FABP4 concentrations did not differ between trials. Additionally, insulin concentrations in the SE trial remained higher both immediately after exercise and post-exercise compared with the LE trials. In a previous study, we examined the effect of increased insulin concentrations due to carbohydrate intake on FABP4 concentration induced by acute aerobic exercise [20]. In that study, despite insulin concentrations being approximately sevenfold higher in the carbohydrate intake trial (~ 30 uU/mL) than in the non-carbohydrate intake trial (~ 4 uU/mL), post-exercise FABP4 concentrations increased similarly in both trials [20]. This suggests that even relatively high insulin concentrations minimally affect the increased FABP4 concentrations induced by acute aerobic exercise. Given that the difference in insulin concentration in this study is much smaller than in our previous study [20], the slight difference in insulin levels is unlikely to significantly impact the difference in FABP4 concentrations between the SE and LE trials in this study.

This is the first study to demonstrate that extended exercise duration augments the increase in circulating FABP4 concentrations. Previous studies have not reported the effects of aerobic exercise lasting > 40 min on FABP4 concentrations [16–18, 20]. The circulating FABP4 concentration in this study was approximately 1.3-fold higher in the LE trial than in the SE trial at 30 and 60 min post-exercise, suggesting that extended exercise duration enhances FABP4 secretion induced by low-intensity exercise. However, we previously reported that the magnitude of the increase in FABP4 concentrations was similar for exercises with matched energy expenditure, despite differences in exercise intensity and duration (40 min for low-intensity [40%VO_2_peak] and 25 min for moderate-intensity [60%VO_2_peak] acute aerobic exercise) [17]. The discrepancy in the magnitude of the increase in circulating FABP4 concentrations between our previous and current study may be explained by differences in the energy expenditure determined by the interrelationship between exercise intensity and duration. In the previous study, energy expenditure was matched between the two exercise conditions to isolate the effect of exercise intensity on circulating FABP4 concentrations [17]. In contrast, in this study, trials with varying exercise durations were designed to examine the effect of exercise duration on circulating FABP4 concentrations, resulting in differing energy expenditures between the SE and LE trials. Additionally, the energy expenditure in the LE trial was markedly higher than in the previous study [17]. Thus, the magnitude of energy expenditure during exercise may be related to the magnitude of FABP4 secretion. Even short-duration, high-intensity exercise with substantial energy expenditure may promote increased FABP4 secretion. In the future, it will be necessary to investigate the influence of energy expenditure on FABP4 secretion, considering the interaction between exercise intensity and duration.

The extent of exercise-induced FABP4 secretion may be related to the accumulation of body fat. The basal circulating FABP4 concentration is correlated with the amount of body fat [30–33]. However, no study has reported a relationship between exercise-induced FABP4 secretion and body fat. Recent studies have shown that baseline circulating FABP4 concentrations remain unchanged in mice with FABP4 deletion in adipocytes in vivo [4], though FABP4 responses to lipolytic stimulation are reduced in these mice [4]. This suggests that adipocytes are the primary source of increased FABP4 concentration due to lipolytic stimulation. Given that the lipolytic rate increases at least 15 min after the onset of acute low-intensity exercise and remains elevated thereafter [19], FABP4 secretion from adipocytes is likely to be continuously stimulated during low-intensity exercise. Therefore, it is possible that the accumulation of body fat mass may contribute to the degree of exercise-induced increases in FABP4 secretion.

The physiological effects of increased circulating FABP4 secretion following acute exercise are not fully understood. Circulating FABP4 increases hepatic glucose production [2, 10]. Since glucose disposal improves following aerobic exercise [34, 35], glucose production in the liver must be increased to maintain a stable blood glucose concentration. Increased circulating FABP4 concentrations following acute exercise may facilitate glucose supply from the liver [2]. Further studies are needed to elucidate the physiological effects of increased circulating FABP4 secretion following acute exercise.

This study had several limitations. First, the participants were young, healthy men; therefore, our findings may not be generalizable to patients with diseases, women, adolescents, and older adults. Second, we did not conduct control trials (no exercise trials). However, we confirmed that FABP4 concentrations did not change after 100 min of fasting [17]. Third, the experimental protocol in this study was only designed for a 60-min postexercise period. Further studies are required to follow long-term changes in circulating FABP4 concentrations.

## Conclusion

This study demonstrated that prolonged exercise in healthy men led to a greater increase in circulating FABP4 concentrations immediately after exercise and post-exercise. Furthermore, the extent of the increase in circulating FABP4 concentrations after acute exercise correlated with body fat accumulation. These findings indicate that exercise duration may be one of the factors accelerating the increase in FABP4 secretion into the circulation. Additionally, body fat accumulation may contribute to increased FABP4 secretion induced by acute aerobic exercise.

## Supplementary Information

Supplementary Material 1: Figure S1. Flowchart of participants.

Supplementary Material 2: Table S1. The Spearman rank correlation coefficients between the changes in FABP4 concentration and the changes in hormones, and metabolites from baseline to immediately after exercise.

Supplementary Material 3: Table S2. The Spearman rank correlation coefficients between the changes in FABP4 concentration and the fat oxidation and percentage of fat oxidation to total energy expenditure during aerobic exercise.

## Data Availability

All data generated or analyzed during this study are included in this published article.

## Abbreviations

FABP4: Fatty acid-binding protein 4
ATGL: Adipose triglyceride lipase
HSL: Hormone-sensitive lipase
VO_2_peak: Peak oxygen uptake
W: Watt
SE trial: A short-duration aerobic exercise + rest trial
LE trial: A long-duration aerobic exercise + rest trial
HR: Heart rate
EDTA: Ethylenediaminetetraacetic acid
Cre: Creatinine
FFA: Free fatty acid
TC: Total cholesterol
HDLC: High-density lipoprotein cholesterol
TG: Triglyceride
HbA1c: Hemoglobin A1c
eGFR: Estimated glomerular filtration rate
EE: Energy expenditure
RER: Respiratory exchange ratio
VO_2_: Oxygen uptake
iAUC: Incremental area under the curve
ES: Effect size
SD: Standard deviation
