# Supplementary figures and images for: Response of circulating fatty acid binding protein 4 concentration to low-intensity acute aerobic exercise is amplified in an exercise duration-dependent manner in healthy men
Source: J Physiol Anthropol. 2024 Dec 20;43:31. doi: 10.1186/s40101-024-00379-y (PMC11660565; doi:10.1186/s40101-024-00379-y)

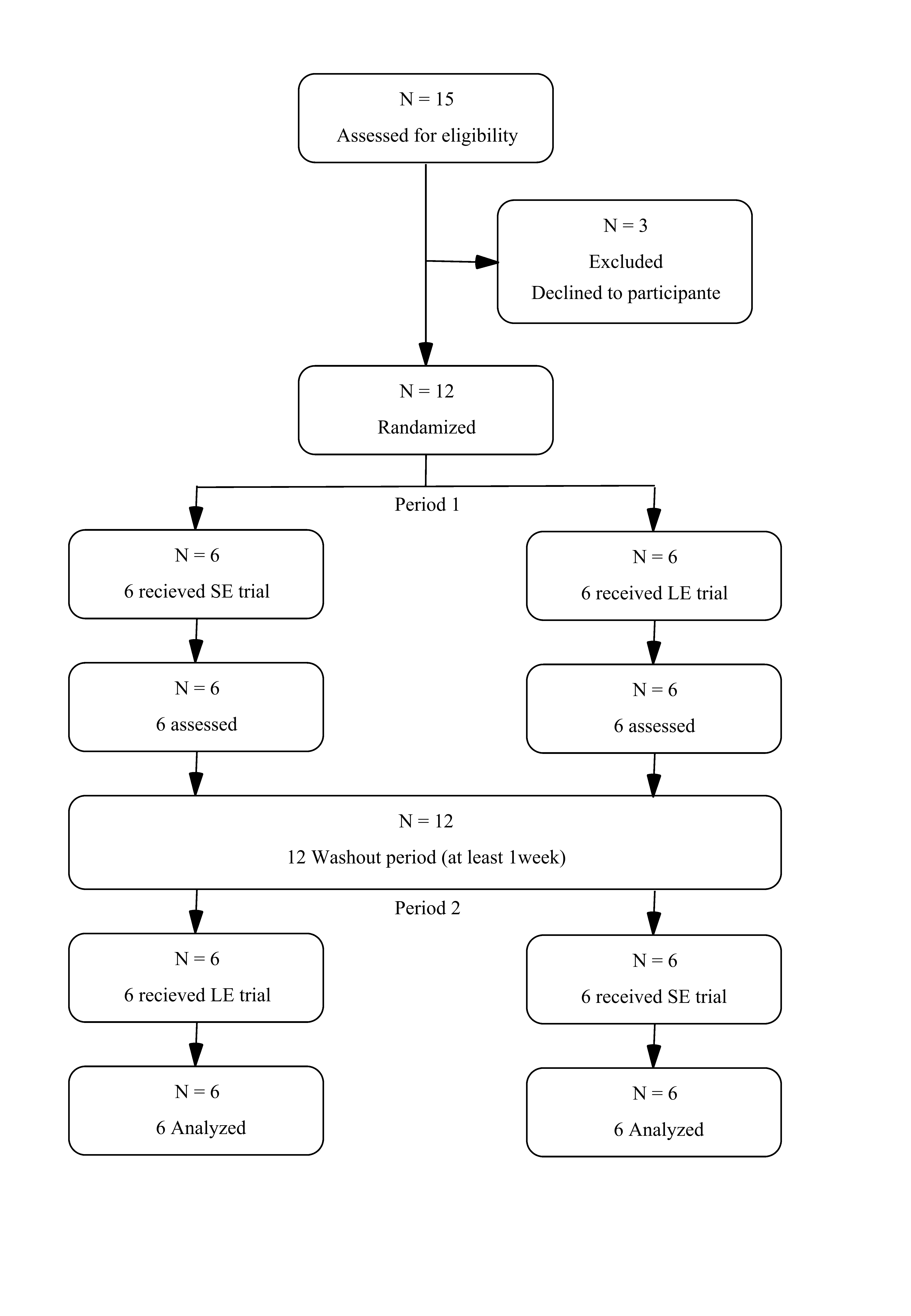

Supplement: Supplementary file 1 — Supplementary Material 1: Figure S1. Flowchart of participants. [file 40101_2024_379_MOESM1_ESM.tiff]
