# Supplementary material for: Response of circulating fatty acid binding protein 4 concentration to low-intensity acute aerobic exercise is amplified in an exercise duration-dependent manner in healthy men
Source: J Physiol Anthropol. 2024 Dec 20;43:31. doi: 10.1186/s40101-024-00379-y (PMC11660565; doi:10.1186/s40101-024-00379-y)
Supplement: Supplementary file 2 — Supplementary Material 2: Table S1. The Spearman rank correlation coefficients between the changes in FABP4 concentration and the changes in hormones, and metabolites from baseline to immediately after exercise. [file 40101_2024_379_MOESM2_ESM.docx]

| Table S1. The Spearman rank correlation coefficients between the changes in FABP4 concentration and the changes in hormones, and metabolites from baseline to immediately after exercise. | | | | | | | | |
| --- | --- | --- | --- | --- | --- | --- | --- | --- |
|  |  | Adrenaline | Noradrenaline | Cortisol | Insulin | Glucose | FFA | Glycerol |
| *SE trial* | |  |  |  |  |  |  |  |
| Baseline to immediately after exercise | r_s_ | 0.259 | -0.007 | -0.098 | 0.035 | 0.126 | -0.427 | -0.217 |
|  | p-value | 0.417 | 0.983 | 0.762 | 0.914 | 0.697 | 0.167 | 0.499 |
| Baseline  to 30 min post-exercise | r_s_ | 0.154 | 0.196 | -0.084 | 0.014 | -0.259 | 0.112 | 0.273 |
|  | p-value | 0.633 | 0.542 | 0.795 | 0.966 | 0.417 | 0.729 | 0.391 |
| Baseline  to 60 min post-exercise | r_s_ | 0.000 | 0.119 | 0.049 | 0.063 | -0.182 | 0.021 | 0.077 |
|  | p-value | 1.000 | 0.713 | 0.880 | 0.846 | 0.572 | 0.948 | 0.812 |
| *LE trial* | |  |  |  |  |  |  |  |
| Baseline to immediately after exercise | r_s_ | -0.259 | -0.077 | 0.161 | 0.413 | 0.266 | 0.315 | 0.175 |
|  | p-value | 0.417 | 0.812 | 0.618 | 0.183 | 0.404 | 0.319 | 0.587 |
| Baseline  to 30 min post-exercise | r_s_ | -0.462 | -0.280 | -0.140 | 0.399 | 0.371 | 0.434 | 0.035 |
|  | p-value | 0.131 | 0.379 | 0.665 | 0.199 | 0.236 | 0.159 | 0.914 |
| Baseline  to 60 min post-exercise | r_s_ | -0.301 | -0.329 | -0.091 | 0.175 | 0.070 | 0.469 | -0.007 |
|  | p-value | 0.342 | 0.297 | 0.779 | 0.587 | 0.829 | 0.124 | 0.983 |
| FFA, free fatty acid; SE, short-duration exercise; LE, long-duration exercise. | | | | | | | | |
