# Supplementary material for: Response of circulating fatty acid binding protein 4 concentration to low-intensity acute aerobic exercise is amplified in an exercise duration-dependent manner in healthy men
Source: J Physiol Anthropol. 2024 Dec 20;43:31. doi: 10.1186/s40101-024-00379-y (PMC11660565; doi:10.1186/s40101-024-00379-y)
Supplement: Supplementary file 3 — Supplementary Material 3: Table S2. The Spearman rank correlation coefficients between the changes in FABP4 concentration and the fat oxidation and percentage of fat oxidation to total energy expenditure during aerobic exercise. [file 40101_2024_379_MOESM3_ESM.docx]

| Table S2. The Spearman rank correlation coefficients between the changes in FABP4 concentration and the fat oxidation and percentage of fat oxidation to total energy expenditure during aerobic exercise. | | | | | |
| --- | --- | --- | --- | --- | --- |
|  |  | Fat oxidation  (g/min) | Fat oxidation  (g/40 min or g/70 min) | Percentage of fat oxidation (%) |  |
| *SE trial* | |  |  |  |  |
| Baseline  to immediately after exercise | r_s_ | 0.095 | 0.056 | 0.119 |  |
|  | p-value | 0.769 | 0.863 | 0.713 |  |
| Baseline  to 30 min post-exercise | r_s_ | -0.106 | -0.126 | -0.161 |  |
|  | p-value | 0.744 | 0.697 | 0.618 |  |
| Baseline  to 60 min post-exercise | r_s_ | -0.222 | -0.231 | -0.203 |  |
|  | p-value | 0.488 | 0.471 | 0.527 |  |
| *LE trial* | |  |  |  |  |
| Baseline  to immediately after exercise | r_s_ | 0.032 | 0.021 | 0.084 |  |
|  | p-value | 0.923 | 0.948 | 0.795 |  |
| Baseline  to 30 min post-exercise | r_s_ | -0.025 | -0.028 | -0.007 |  |
|  | p-value | 0.940 | 0.931 | 0.983 |  |
| Baseline  to 60 min post-exercise | r_s_ | -0.102 | -0.112 | -0.147 |  |
|  | p-value | 0.753 | 0.729 | 0.649 |  |
| SE, short-duration exercise; LE, long-duration exercise. | | | | | |
